# Assessing advanced handwritten text recognition engines for digitizing historical documents

**DOI:** 10.1007/s42803-025-00100-0

**Published:** 2025-05-12

**Authors:** C. A. Romein, A. Rabus, G. Leifert, P. B. Ströbel

**Affiliations:** 1 Huygens Institute for the History and Culture of the Netherlands, Amsterdam, the Netherlands; 2 READ-COOP SCE, Innsbruck, Austria; 3 Walter Benjamin Kolleg, University of Bern, Bern, Switzerland; 4 University of Twente, Enschede, the Netherlands; 5 Albert-Ludwigs-Universität Freiburg, Freiburg Im Breisgau, Germany; 6 Planet AI, Rostock, Germany; 7 University of Zürich, Zurich, Switzerland

**Keywords:** Handwritten text recognition (HTR), Language models, Historical document digitization, Multilingual content, Digital humanities, TrOCR, PyLaia, IDA, Transkribus, Planet AI

## Abstract

This study provides critical insights and evaluates the performance of state-of-the-art Handwritten Text Recognition (HTR) engines—PyLaia, HTR + , IDA, TrOCR-f, and Transkribus’ proprietary Transformer-based “supermodel” Titan—to digitize historical documents. Using a diverse range of datasets that include different scripts, this research assesses each engine's accuracy and efficiency in handling multilingual content, complex styles, abbreviations, and historical orthography. Results indicate that, while all engines can be trained or fine-tuned to improve performance, Titan and TrOCR-f exhibit superior out-of-the-box capabilities for Latin-script documents. PyLaia, IDA, and HTR + excel in specific non-Latin scripts when specifically trained or fine-tuned. This study underscores the importance of training, fine-tuning, and integrating language models, providing critical insights for future advancements in HTR technology and its application in the digital humanities.

## Introduction

Handwritten Text Recognition (HTR) technology, a transformative capability in the realm of digital humanities, enables the transcription and analysis of historical handwritten documents with unprecedented accuracy and efficiency (Leifert et al., 2023, 2024). This capability is a testament to ongoing innovations in machine learning as an application within artificial intelligence, is particularly significant for researchers aiming to unlock the vast troves of information embedded within archival materials. The conversion of handwritten texts into machine-readable formats, facilitated by HTR, enhances access, interpretation, and preservation of historical records, thereby contributing to a deeper understanding of our collective past (Nockels et al., 2024). The advent of HTR technology has revolutionized the field of historical research, allowing scholars to process large volumes of handwritten texts that were previously inaccessible or too labor-intensive to analyze manually. This technological breakthrough accelerates the pace of research and opens up new avenues for interdisciplinary studies, including linguistics, history, and cultural studies. As a result, the ability to accurately transcribe and analyze historical documents has become a cornerstone of modern digital humanities (Pinche & Stokes, 2024).

Despite the significant progress made in HTR technology, the performance of various HTR engines can vary widely depending on the nature of the text being processed. Factors such as language, script, orthography, and the physical condition of the documents play a crucial role in determining the accuracy and efficiency of these engines (Ehrmann et al., 2023). Therefore, a comprehensive evaluation of HTR engines is essential to identify their strengths and limitations and to guide future developments in this field.

The primary objective of this study is to evaluate the performance of state-of-the-art HTR engines, specifically PyLaia, HTR +, Intelligent Document Analysis (IDA), and two versions of TrOCR (Li et al., 2021): TrOCR-f—fine-tuned on the datasets mentioned below, and Transkribus’ proprietary Transformer-based “supermodel” Titan, on diverse datasets.^1^ The engines PyLaia and Titan have been integrated into the Transkribus platform (Muehlberger et al., 2019), a widely utilized document transcription and analysis tool; IDA is a proprietary engine developed by Planet AI. TrOCR-f is the fine-tuned version of Microsoft’s Transformer-based OCR solution. The datasets employed in this evaluation encompass a broad range of languages, scripts, and historical contexts, thereby providing a robust benchmark for assessing the capabilities of each HTR engine.

This study addresses the following research question: How do state-of-the-art HTR engines perform when applied to diverse historical datasets, and what are their relative strengths and weaknesses regarding transcription accuracy and efficiency? Four distinct datasets were selected to address this question, each presenting unique challenges related to language, script, and historical context. These datasets include:

1. **Roman Type Print:** Printed texts in Dutch and French from the seventeenth century, characterized by historical orthography and multilingual content (*KBNLresearch/EntangledHistories*, 2019/2019; Romein et al., 2020b).
2. **Republic(7)**^2^**:** Documents from the Dutch States General resolutions written between 1576 and 1795, featuring formal historical language and detailed resolution records (Sluijter et al., 2023).
3. **Glagolitic:** Texts written in the Glagolitic script, a Slavic script formerly used in Croatia, known for its continuous script, ligatures, and numerous abbreviations (Rabus & Thompson, 2023).
4. **Shorthand:** German shorthand texts, specifically"Deutsche Einheitskurzschrift"(DEK), involve complex one-to-many relationships between shorthand symbols and longhand transcriptions.

This study's objective is to provide valuable insights into the capabilities of current HTR technology by systematically analyzing the performance of PyLaia, HTR +, IDA, TrOCR-f, and Titan across a series of datasets. The findings will inform the selection of appropriate HTR engines for specific transcription tasks and highlight areas where further improvements are needed. Ultimately, this research contributes to the ongoing development and refinement of HTR technologies, enhancing their utility for the digital humanities and beyond.

## Significance of HTR technology

The impact of HTR technology extends beyond mere transcription, catalyzing the digital transformation of cultural heritage. By rendering historical documents searchable and analyzable, HTR technology amplifies scholarly research, broadens access to information, and nurtures collaboration. It creates a platform for historians, linguists, and computer scientists to converge and pioneer new frontiers in their respective fields, fostering a sense of shared responsibility and collective progress in interdisciplinary research.

Despite its transformative potential, HTR technology faces significant challenges, mainly when applied to historical documents (Romein et al., 2020a, b, c). These challenges include diverse handwriting styles, deteriorated manuscript conditions, multilingual content, and archaic or non-standard orthography. Addressing these hurdles requires sophisticated algorithms and the combined expertise of researchers, scholars, and professionals in digital humanities, history, linguistics, and computer science. This intricate landscape illustrates the ongoing necessity for refining and enhancing HTR technology, with the clear implication that continued contributions are paramount.

## Methodology

The methodology section of this study outlines the approach taken to evaluate the performance of five selected HTR engines: PyLaia, HTR +, IDA, TrOCR-f, and Titan. This evaluation utilized a series of datasets encompassing various languages, scripts, and historical contexts. The methodology ensures a rigorous and comprehensive assessment of each HTR engine's capabilities, providing valuable insights into its strengths and weaknesses. Four distinct datasets were selected to provide a robust benchmark for evaluating the HTR engines, each representing a unique combination of language, script, and historical context challenges. We recognize that our evaluation criteria would benefit from deeper theoretical contextualization within the broader landscape of HTR assessment. The experimental procedure, while designed to provide comparative insights across engines, has inherent limitations in terms of cross-engine standardization and parameter optimization. A particularly noteworthy finding emerged regarding the impact of language models on recognition performance across the tested engines. This variable significantly influenced results in ways that suggest language model integration will be a critical consideration for future HTR development and deployment scenarios. Future work should further explore the theoretical underpinnings of evaluation metrics in relation to specific use cases and provide more granular analysis of how language models interact with different document characteristics to enhance recognition accuracy.

### Datasets

#### Roman type print

**Fig. 1:**
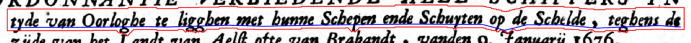
Example of Roman type print

This dataset comprises printed texts in Dutch and French from the seventeenth century, rendered in the"Roman"font (see: Fig. 1). The font was commonly used for books of ordinances starting in the early seventeenth century (Romein, Veldhoen, et al., 2020a).

Volume:

- Words: 88,105
- Characters: 132,866
- Lines: 3,255
- Pages: 41

Challenges:

- Multilingual content (Dutch and French) within the same document, sometimes on the same page.
- Historical orthography, which may differ significantly from modern usage.

The model for this dataset was trained using this relatively small dataset due to the limited availability of printed text from this period. The primary challenge was the multilingual nature of the content, which required the model to switch between Dutch and French seamlessly.

#### Republic(7)

**Fig. 2:**
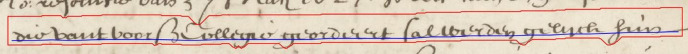
Example of the handwriting of the republic sources

This dataset consists of documents from the Dutch States General resolutions written between 1576 and 1795 (See: Fig. 2). These documents have been transcribed as part of the Republic! project at the Huygens Institute (*Republic*, n.d.).

Volume:

- Words: 236,349
- Characters: 1,566,028
- Lines: 42,359
- Pages: 465

Challenges:

- Formal historical language
- Detailed resolution records

The extensive training set for this model ensures a robust dataset for training purposes. The ground truth (GT) has been published on Zenodo (Sluijter et al., 2023). The primary challenge lies in these resolutions'formal and detailed language.

#### Glagolitic

**Fig. 3:**
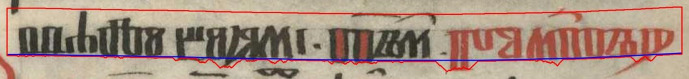
Example of Glagolitic script

This dataset features texts written in the Glagolitic script, a Slavic script used in Croatia characterized by scriptura continua, where no spaces exist between words (See: Fig. 3).

Volume:

- Words: 397,198
- Characters: 2,125,155
- Lines: 68,229
- Pages: 1,146

Challenges:

- Continuous script without word divisions
- Numerous abbreviations

The Glagolitic script was developed for Old Church Slavonic in the ninth century and continued in Croatia through the Middle Ages. The transcription adheres to the Croatian philological tradition, employing the Latin script with diacritics. The primary challenge for this dataset is the prevalence of abbreviations in the manuscripts, which are expanded in the transcriptions with parentheses indicating omitted letters. The model must effectively learn to resolve these abbreviations.

#### Shorthand

**Fig. 4:**
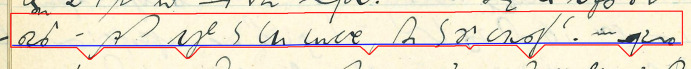
Example of German shorthand (Deutsche Einheitskurzschrift/DEK)

This dataset contains German shorthand texts, specifically "Deutsche Einheitskurzschrift" (DEK) (See: Fig. 4).

Volume:

- Words: 144,709
- Characters: 941,450
- Lines: 19,010
- Pages: 698

Challenges:

- One-to-many relationships between shorthand symbols and their longhand transcriptions

The dataset is particularly complex due to the shorthand notation, where a single shorthand symbol may correspond to multiple longhand transcriptions. Consequently, the models face difficulties achieving low error rates, with Character Error Rates (CERs) between 10 and 20% being common (Holley 2009). The effectiveness of language models is crucial for improving model performance in this context.

### Textline extraction

The polygons of the Transkribus ground truth frequently omit essential components of the line images. Consequently, we decided to recalculate the polygons utilizing the existing baselines. A Planet AI polygon generator was employed for all text line extraction on all training sets, ensuring that Titan, TrOCR, and IDA operate on the same text lines. In the case of Shorthand and Glagolitic, the validation set contains 500 single lines where the polygon generator fails. The original polygons are utilized for these lines.

### HTR engines and their architectures

The HTR engines selected for this study employ different architectural approaches (see Table 1):

**Table 1:** Engines and their specifications

Engine	Augmentation	Model architecture	LM type	Core
HTR +	yes	CNN-LSTM	Regex & Word Unigram	Tensorflow 1
PyLaia	yes (Tarride et al., 2024)	CNN-LSTM(5)	Character-Ngram (*N* > = 5)	Pytorch
IDA	yes, massive	CNN-Conformer	Character-5-g	Tensorflow 2
TrOCR	no	Transformer	Transformer	Pytorch

^a^The model has not been fine-tuned towards our datasets see “Methods” for more information

#### PyLaia

PyLaia is an HTR engine that employs a Convolutional + Neural Network LSTM architecture, rendering it adept at processing data sequences (Puigcerver, 2017/2024). This combination allows the engine to recognize and transcribe handwritten text by capturing spatial and temporal features.

Integrated into the Transkribus platform, PyLaia is designed to be user-friendly and requires no programming skills for model training and inference. This accessibility makes it a popular choice among researchers who seek a balance between ease of use and high performance.

PyLaia is a robust HTR engine that incorporates sophisticated architecture with accessible integration options. Its design is tailored to researchers from diverse fields, enabling them to harness sophisticated transcription technology without requiring extensive technical expertise.

#### HTR + 

HTR + was developed as a joint work from Planet AI and the University of Rostock. The HTR engine is similar to PyLaia. The main difference is the improved line extraction algorithm to clean up and normalize the line image for size, line skew or slant, background noise and other artifacts. The engine is based on Java, OpenCV, and TensorFlow. The speed of training or applying the HTR + engine is comparable with PyLaia.

Historically, HTR + was also integrated into the Transkribus platform and thus easy for clients to use. Due to license issues, it was removed from Transkribus and is no longer available.

#### IDA

The IDA system developed by Planet AI is an improved and enhanced version of HTR +. It uses a convolutional architecture stacked with either an LSTM or conformer architecture (Gulati et al., 2020), depending on the quality and speed required. Both architectures are tested for the four datasets. Whereas the LSTM architecture leads to similar results as PyLaia and HTR +, the more complex conformer architecture leads to longer training and slower inference time, but better quality. After training, transcriptions within Planet AI's commercial product suite can be completed without requiring expert knowledge.

#### TrOCR

TrOCR, initially developed by Microsoft, is based on the Vision and LLM-Transformer architecture (Li et al., 2021). It combines vision capabilities with large language models for highly effective handwritten text recognition.

The TrOCR-f models were prepared in Bern and Zurich, building upon Microsoft's foundational architecture. TrOCR was fine-tuned on the datasets used for this paper. However, using TrOCR-f necessitates programming skills and decent hardware for fine-tuning, rendering it more suitable for users with technical expertise in AI.

Titan (also known as Text Titan) was created by Transkribus, utilizing Microsoft's underlying TrOCR technique. Titan is fine-tuned on a vast corpus of historical documents across multiple languages, integrated into the Transkribus platform and designated as a"supermodel,"Titan is designed for straightforward usability, offering exceptional performance on Latin-script documents without additional fine-tuning.

By leveraging the Vision + LLM-Transformer architecture, both TrOCR-f and Titan provide powerful solutions for handwritten text recognition, catering to diverse user needs and levels of expertise.

## Evaluation metric

The primary metric for evaluating the HTR engines is the Character Error Rate (CER). CER is calculated using the following steps:

1. **Levenshtein Distance**: Calculate the distance between the transcribed text (hypothesis) and the ground truth.
2. **Normalization**: Normalize the error count by dividing by the total number of characters in the ground truth.
3. **CER Calculation**:
  $$CER\text{=}\frac{Levenshtein \, Distance \, (Errors)}{Total \, Characters \, \in \, Ground \, Truth}$$

### Criteria for evaluation

A) **Case Sensitivity**: Evaluating CER in a case-sensitive manner is essential, though upper-/lowercase correspondences can pose challenges for historical data. When time permits, a case-insensitive evaluation can be considered to account for these inconsistencies.
B) **Unicode Normalization**: Ensuring consistency using Unicode Normalization Form C (NFC) in all comparison methods is crucial. Differences in normalization can affect the comparison results, leading to inaccuracies (*UAX #15: Unicode Normalization Forms*, n.d.-a).
C) **Character Classes**: Some models have advanced capabilities, such as adding quotation marks for direct speech without visual cues. Evaluating CER separately for different character classes, such as letters and punctuation, is beneficial. Using resources, you can filter all strings by categories (Compart AG, n.d.).
D) **Word Error Rate (WER)**: While CER is indispensable, WER is an optional but interesting metric. For instance, PyLaia with a language model (LM) shows significantly lower WER than HTR + with an LM, indicating its normative approach to word recognition. This aspect warrants both quantitative and qualitative analysis. Normative models might benefit historians but could be less favorable for philologists.
E) **Word Separation Errors**: Word separation errors should be penalized. Evaluating a category without punctuation and spacing characters can provide insights into the model's handling of word separations.

Considering these criteria, we can obtain a comprehensive and nuanced evaluation of the HTR engines'performance across different aspects of text recognition.

## Experimental procedure

We primarily focused on the Character Error Rate (CER) to compare the different HTR engines because a character sequence can be tokenized into words. For example, the sequence"It's hard."could be separated into two (space-tokenizer) or five (category-tokenizer) words, depending on the interpretation of a word. Given the different approaches of the HTR engines, they exhibit varying strengths and weaknesses in character recognition. We evaluated CER by filtering the ground truth and hypothesis characters to uncover these strengths and weaknesses. For simplification, we filter by Unicode character categories as referenced in Unicode General Category Values (*UAX #44: Unicode Character Database*, n.d.). As general normalization, we map “¬”, denoting hyphenation when the hyphenated word occurs at the end of a line, to “-” and < TAB > to < SPACE > and apply the Normal Form C (NFC) as detailed in Unicode Normalization Forms (*UAX #15: Unicode Normalization Forms*, n.d.-b). Additionally, all duplicate spaces and leading and trailing spaces are removed.

### Data preparation

Each dataset was preprocessed to standardize the text format and ensure consistency in evaluation (see: Table 2). Specific normalization procedures included mapping special characters (e.g., “¬” to “-” and < TAB > to < SPACE >), applying Unicode normalization (NFC), and removing duplicate spaces and leading/trailing spaces.

**Table 2:** Normalisations

Name	Modification	Example
Original	None	"in Paris (it’s a capital)."
base	As described above	"in Paris (it’s a capital)."
alnum	Only keep L, N, Z, M categories	"in Paris its a capital"
lower	Apply.toLower()	"in paris (it’s a capital)."
nospace	Remove Z category	"inParis(it’sacapital)."
alnumlower	Combine alnum and lower	"in paris (its a capital)."

Data Preparation: all models need a preprocessing of the text line. The models Pylaia, HTR + and IDA use similar methods to normalize the textline, like size normalization, contrast enhancement, and cropping.

### Training and inference

Each HTR engine was trained using the respective training sets from the datasets:

- For PyLaia and HTR +, training was conducted using the Transkribus GUI and the default settings, simplifying the process for users without programming skills.
- IDA: All models were trained from the ground up by an employee of Planet AI. The training regimen was uniformly applied across all models, beginning with a ramp-up of the learning rate over the initial 10 epochs. This was subsequently followed by an exponential decay in the learning rate. To further refine the models, an additional cosine decay was employed over the final 50 epochs of training. Furthermore, the language model (LM) employed was independently trained using the training dataset.
- We fine-tuned dedicated TrOCR-f models on the pre-trained large handwritten TrOCR as released by Li et al. (2021).^3^ We experimented with three, five and ten epochs and found that for all datasets mentioned above, models fine-tuned for five epochs performed best. We used one epoch of warmup to adapt the model to the new data. Titan is a TrOCR model fine-tuned on a massive training set compiled from documents on Transkribus. In contrast to all other TrOCR-based models, the model is directly usable within the Transkribus platform, so no extra training or fine-tuning is necessary.

### Evaluation

Each model's CER was calculated for base and alphanumeric filtered transcriptions. Where possible, additional language models (LM) were applied to engines to enhance performance, and the impact of these LMs on CER was evaluated.

**Evaluation types:**

- **CER:** Texts are normalized using NFC—CHECK—> base.
- **CER_NoSpace:** Like CER, but without the Z category—CHECK—> nospace.
- **CER_lower:** Like CER, but all lowercase—CHECK—> lower.
- **CER_ALNUM:** Like CER but only L, N, M categories—CHECK—> alnum, but with Z/Space category.

This comprehensive evaluation procedure ensures a fair comparison of the HTR engines across various dimensions of text recognition, providing insights into their performance and areas for improvement.

## Results

### Results organized per model

For each dataset, we calculate the CER for each model. This results in the following tables (see Tables 3, 4, 5, and 6).

**Table 3:** CER Base

Dataset	Roman type print		Republic (7)		Glagolitic		Shorthand	
Engine	w/o LM	w/LM	w/o LM	w/LM	w/o LM	w/LM	w/o LM	w/LM
HTR +	1.74%		**1.78%**			5.65%		13.62%
PyLaia	2.26%	1.92%	3.79%		6.32%	6.05%	11.90%	11.18%
IDA	2.78%	2.67%	3.54%	3.05%	4.83%	**4.54%**	9.53%	**9.29%**
TrOCR-f	4.74%		3.24%		4.67%		9.70%	
Titan^a^	**1.50%**		3.61%

**Table 4:** CER Alnum

Dataset	Roman type print		Republic (7)		Glagolitic		Shorthand	
Engine	w/o LM	w/LM	w/o LM	w/LM	w/o LM	w/LM	w/o LM	w/LM
HTR +	1.51%		**1.31%**			3.92%		13.45%
PyLaia	1.77%	1.51%	2.90%		4.77%	4.42%	11.75%	10.93%
IDA	2.40%	2.29%	2.76%	2.36%	3.14%	**2.92%**	9.36%	**9.10%**
TrOCR-f	4.37%		2.83%		3.64%		9.75%	
Titan*	**1.10%**		2.80%

**Table 5:** CER-Nospace

Dataset	Roman type print		Republic (7)		Glagolitic		Shorthand	
Engine	w/o LM	w/LM	w/o LM	w/LM	w/o LM	w/LM	w/o LM	w/LM
HTR +	1.49%		**1.95%**		5.52%		14.89%	
PyLaia	2.08%	1.77%	4.10%		6.19%	6.08%	12.95%	12.61%
IDA	2.39%	2.58%	3.17%	3.28%	4.71%	4.39%	**10.06%**	**10.06%**
TrOCR-f	3.15%		4.20%		**3.80%**		10.10%	
Titan*	**1.39%**		3.27%

**Table 6:** CER-Alnumlowernospace

Dataset	Roman type print		Republic (7)		Glagolitic		Shorthand	
Engine	w/o LM	w/LM	w/o LM	w/LM	w/o LM	w/LM	w/o LM	w/LM
HTR +	1.16%		1.35%		3.41%		13.40%	
PyLaia	1.40%	1.40%	2.65%	2.61%	4.36%	4.10%	11.66%	11.66%
IDA	2.04%	2.04%	2.41%	**2.21%**	2.61%	2.39%	**8.83%**	**8.83%**
TrOCR-f	2.61%		2.93%		**2.43%**		9.57%	
Titan*	**0.82%**		2.56%					

### Results organized per dataset

For each dataset, we calculate the CER for each type of text. This results in the following tables (see: Tables 7, 8, 9 and 10).

#### Roman type print

**Table 7:** Results of Roman type print

Network	base	alnum	nospace	alnumlowernospace
htr- +	1.74	1.51	1.49	1.16
ida	2.78	2.40	2.39	2.04
ida_lm	2.67	2.29	2.58	2.04
pylaia	1.92	1.77	2.08	1.40
titan	1.50	1.10	1.39	0.82
trocr-f	4.74	3.24	3.15	2.61

#### Republic(7)

**Table 8:** Results of Republic

Network	base	alnum	nospace	alnumlowernospace
htr- +	1.78	1.31	1.95	1.35
ida	3.54	2.76	3.17	2.41
ida_lm	3.05	2.36	3.28	2.21
pylaia	3.89	2.90	4.10	2.65
titan	3.61	2.80	3.72	2.56
trocr-f	3.97	3.15	4.20	2.93

#### Glagolitic

**Table 9:** Results of glagolitic

Network	base	alnum	nospace	alnumlowernospace
htr- + _lm	5.65	3.92	5.52	3.41
ida	4.83	3.16	4.71	2.62
ida_lm	4.54	2.92	4.39	2.39
pylaia	6.32	4.77	6.19	4.36
pylaia_lm	6.05	4.42	6.08	4.10
trocr-f	4.67	3.64	3.80	2.43

#### Shorthand

**Table 10:** Results of shorthand

Network	base	alnum	nospace	alnumlowernospace
htr- + _lm	13.62	13.45	14.89	13.40
ida	9.53	9.19	10.06	8.83
ida_lm	9.29	9.10	10.06	8.83
PyLaia	11.90	11.75	12.96	11.66
PyLaia_lm	11.18	11.75	12.61	11.66
trocr-f	9.70	9.75	10.10	9.57

## Comparison and analysis

The final step involved comparing the performance of the HTR engines across the different datasets. This comparison focused on identifying each engine's specific strengths and weaknesses in handling the diverse challenges presented by the datasets. The following key factors were considered in the analysis:

- The impact of dataset characteristics (e.g., script complexity, language diversity) on CER.
- The performance differences between base and alnum-filtered transcriptions.
- The benefits and limitations of incorporating an additional language model.

### Roman type print

The Roman dataset, which includes printed texts in Dutch and French from the seventeenth century, presents unique challenges due to historical orthography and multilingual content. To ensure fair comparison across engines that may employ different character sets, we applied consistent normalization techniques in our evaluation methodology. When evaluated under these standardized conditions, Titan exhibits the best base performance with a Character Error Rate (CER) of 1.50%.

Beyond evaluation methodology, we also examined how technical enhancements affect actual performance. The integration of language models genuinely improves results across all engines. TrOCR-f and the IDA variants show moderate performances in their base configurations, with IDA variants specifically demonstrating measurable improvements when language models are incorporated. Notably, Titan achieves the lowest CER of 0.82%, demonstrating its superior capabilities for Latin-script documents from this period. Overall, our analysis of the Roman dataset highlights both the importance of standardized evaluation methods and the genuine performance benefits of language model integration or, for that matter, Transformer technology, in HTR engine optimization.

### Republic (7)

The Republic dataset consists of documents from the Dutch States General resolutions written between 1576 and 1795. The texts in this dataset are characterized by formal historical language and detailed resolution records. In this context, HTR + demonstrates the best base performance with a CER of 1.78%. This can be explained by the workflow of the ground truth data generated: An HTR + model, trained on similar data, was used to create a first suggestion. This was then manually corrected by an expert. Since the corrector likely missed correcting some errors, these errors remain in the ground truth. This means that the trained model is already biased towards HTR +. PyLaia and its variants perform reasonably well, especially with significant improvements when using alphanumeric filtering. The Titan engine maintains strong performance, particularly when alnumlowernospace normalization is applied, resulting in a CER of 2.56%. The findings indicate that language models are essential for enhancing the recognition accuracy of formal historical texts.

### Glagolitic

The Glagolitic dataset features texts written in the Glagolitic script, characterized by scriptura continua, ligatures and numerous abbreviations. The IDA engine shows the best base performance with a CER of 4.83%, and significant improvements are observed with alphanumeric filtering, especially for ida_lm, which achieves a CER of 2.92%. For PyLaia and its variants, this dataset seems to be a more significant challenge, indicating that advanced, ‘smart’ functions in HTR such as the resolution of abbreviations benefit from a more complex neural network architecture. The TrOCR-f engine also performs well, notably when non-alphanumeric characters are excluded, achieving a CER of 2.43%. These results suggest that different model architectures and language models play a role when evaluating HTR engine performance on complex scripts like Glagolitic.

### Shorthand

The Shorthand dataset exhibits intricate one-to-many relationships between shorthand symbols and longhand transcriptions. IDA generally performs well among the evaluated engines, with ida_lm demonstrating the most robust base performance at a CER of 9.29%. The incorporation of language models enhances performance, reducing errors by approximately 0.1 to 0.4 percentage points, which is in line with previous research (Tarride et al., 2024). The PyLaia engines exhibited higher error rates, indicating the complexity of shorthand transcription for these models. These results show that more sophisticated model architectures cope better with highly complex HTR tasks, but emphasize the need for further advancements in HTR and language model technology to handle the intricacies of shorthand transcriptions effectively.

***

The evaluations indicate that the Transformer-based Titan engine offers good overall performance, albeit restricted to Latin-script texts. This demonstrates strong out-of-the-box capabilities and no need for additional training for such use cases. Fine-tuning Microsoft’s Transformer on our datasets (TrOCR-f) also proves effective, especially for complex non-Latin use cases, i.e., Glagolitic and shorthand, where it surpasses PyLaia. Interestingly, fine-tuning the Latin-script datasets did not yield better results than the specific PyLaia models. While PyLaia and its variants perform well when trained on specific Latin and non-Latin script data and are the go-to candidate for easy training within the Transkribus platform, closed-source engines with more complex model architectures like IDA show better performance across multiple datasets overall. Language models consistently enhance the accuracy of HTR engines across all datasets, underscoring their importance in optimizing transcription performance. The study highlights the ongoing challenges in shorthand recognition, suggesting that further technological advancements are necessary to improve transcription accuracy in this area. These findings emphasize the necessity of developing, selecting, training, and fine-tuning HTR engines based on the specific characteristics of the dataset. They also highlight the critical role of language models and advanced normalization techniques in enhancing the performance and accuracy of HTR technologies, ultimately bridging the gap between historical manuscripts and modern digital analysis.

## Conclusion

HTR technology represents a significant advance in digitizing historical texts, offering unparalleled opportunities for research and preservation. This study’s comparative analysis of PyLaia, IDA, TrOCR-f, and Titan provides valuable insights into their respective capabilities, guiding future advancements in this critical area of research. As HTR technology continues to evolve, it promises to further bridge the gap between historical manuscripts and modern digital analysis, enriching our understanding of the past.

It should be noted that the Titan engine has not been fine-tuned on non-Latin script data and cannot be fine-tuned by users of Transkribus, which explains its inability to recognize Glagolitic and Shorthand scripts. Notwithstanding, all the HTR engines utilized in our experiments, namely PyLaia, IDA, TrOCR-f, and Titan as well as Transkribus’ now defunct legacy engine HTR +, demonstrated the capacity to address the various challenges presented by the datasets, including the presence of multiple languages, challenging handwriting, abbreviations, and stenography. These engines can be trained or fine-tuned to enhance their performance further.

Regarding specific datasets, HTR + outperforms PyLaia on Glagolitic, while PyLaia shows comparatively better results on Shorthand. This discrepancy may be attributed to an inherent limitation of HTR +, which is prone to misalignment with the probability matrix when subjected to large subsampling of images. While HTR +, PyLaia, and IDA require well-defined surrounding polygons for accurate recognition, TrOCR-f and Titan, which have been pre-trained and fine-tuned on unmasked text lines, are unaffected by defective polygons.

Incorporating a language model (LM) consistently enhances the performance of HTR +, PyLaia, and IDA. Training these engines from scratch reveals notable differences in training duration. IDA required approximately 48 h on a single GPU to achieve optimal performance on the Republic (7) dataset, whereas PyLaia took only 9 h and 22 min on the Transkribus servers. Fine-tuning of TrOCR-f on a few thousand lines is relatively quick, as evidenced by the 10-h fine-tuning on approximately 40,000 lines from the Republic (7) dataset on an NVIDIA V100 GPU.

The Transformer-based models Titan and TrOCR-f perform exceptionally well for Latin-script handwritten data, likely due to their pre-training on vast corpora that predominantly feature Latin script documents. This inherent bias in training data gives these models a natural advantage when processing Latin-based texts. Titan is effective in its default configuration, benefiting from extensive pre-training that has already optimized its parameters for common Western languages and scripts. Meanwhile, TrOCR-f needs to be fine-tuned because its base model, while powerful, requires adaptation to specific document characteristics and script variations to reach optimal performance. This fine-tuning process allows the model to adjust its attention mechanisms to focus on the distinctive visual features of particular historical documents or handwriting styles. Titan achieves the best performance for early Latin script printings without additional training, again reflecting the advantages conferred by its comprehensive pre-training regimen. TrOCR-f shows lower Character Error Rates (CERs) when fine-tuned on rare and complex scripts than HTR + and PyLaia, demonstrating that transformer architectures can effectively adapt to different types of data. The significant improvement in CER for the Glagolitic dataset when comparing base and alnum can be attributed to the models'difficulty expanding abbreviations and adding correct parentheses. By excluding these characters, the computed CER decreases—the token-based architecture of TrOCR-f results in a smaller improvement. PyLaia offers easy training on Transkribus without requiring programming skills, while TrOCR is freely available but necessitates programming expertise and decent hardware. IDA remains a closed-source solution provided by a company. IT experts manually train TrOCR-f and IDA without hyperparameter optimization.

The CER goes down when ignoring non-alphanumeric characters, such as punctuation and symbols. However, TrOCR-f's CER for Shorthand slightly increases, indicating the model’s proficiency in recognizing punctuation and symbols in this dataset.

Comparing the Transformer-based models, Titan and TrOCR-f, on the Republic (7) dataset shows that TrOCR-f shows good performance in CER-base but is slightly less effective in CER- alnum. Titan has likely benefitted from training on a large and diverse Latin-script historical language corpus. In contrast, punctuation recognition is more dataset-specific and challenging to learn consistently across diverse datasets.

In summary, this study highlights the capabilities and limitations of various HTR engines, emphasizing the importance of model architecture, training, fine-tuning, and the role of additional language models in enhancing performance. Our findings reveal important trade-offs between different architectural approaches: transformer-based models like TrOCR-f and Titan demonstrate superior performance across diverse materials and may eliminate the need for line segmentation, but this comes at the cost of requiring massive training datasets. This often limits digital humanities practitioners to either fine-tuning existing models or using pre-trained models with minimal adaptations. In contrast, sequential LSTM/CTC-based architectures offer the advantage of being trainable on comparatively smaller datasets while still producing valuable results, making them more accessible for domain-specific applications with limited training resources. The findings underscore the need for ongoing development and refinement of HTR technology to meet the diverse demands of historical text recognition, with particular attention to balancing performance requirements against practical constraints of data availability and computational resources.

## Data Availability

Due to copyright, only the dataset of Republic(7) can be found online: see: Sluijter, R., van Koert, R., Baars, M., Swüste, M., van Gent, M., van Gelder, E., Hollestelle, J., Ruigrok, G., Nijenhuis, I., & Oddens, J. (2023). REPUBLIC PageXML ground truth handwritten resolutions States General (Version 1.0) [Dataset]. Zenodo. 10.5281/zenodo.7695131.

## Footnotes

1 For a variety of reasons, other HTR engines, especially Kraken, could not be taken into account for this paper. For a comparison of Kraken and the Transkribus engines, see Rabus & Thompson (2023).

2 This number 7 stands for the model that was created by the Republic team and on which corpus(-part) it was based, since we could use there GT-corpus, we decided to refer to this corpus as Republic(7).

3 See https://huggingface.co/microsoft/trocr-large-handwritten (Retrieved 30 September 2024) .

## References

[CR1] Compart AG, C. (n.d.). *Finde alle Unicode Zeichen von Hieroglyphen bis zu Glyphen – Unicode Compart*. https://www.compart.com/de/unicode/category. Retrieved 30 September 2024, from https://www.compart.com/de/unicode/category

[CR2] *CRediT*. (n.d.). CRediT. Retrieved 20 October 2022, from https://credit.niso.org/

[CR3] Ehrmann, M., Hamdi, A., Pontes, E. L., Romanello, M., & Doucet, A. (2023). Named entity recognition and classification in historical documents: A survey. *ACM Comput. Surv.,**56*(2), 1–27.

[CR4] Gulati, A., Qin, J., Chiu, C.-C., Parmar, N., Zhang, Y., Yu, J., Han, W., Wang, S., Zhang, Z., Wu, Y., & Pang, R. (2020). *Conformer: Convolution-augmented Transformer for Speech Recognition* (No. arXiv:2005.08100). arXiv. 10.48550/arXiv.2005.08100

[CR5] Holley, R. (2009). How good can it get?: Analysing and improving OCR accuracy in large scale historic newspaper digitisation programs. *D-Lib Magazine*, *15*(3/4). 10.1045/march2009-holley

[CR6] *KBNLresearch/EntangledHistories*. (2019). [Jupyter Notebook]. National Library of the Netherlands / Research. https://github.com/KBNLresearch/EntangledHistories (Original work published 2019)

[CR7] Leifert, G., Romein, C. A., Rabus, A., Ströbel, P. B., Kiessling, B., & Hödel, T. (2023). *Evaluating State‐of‐the‐Art Handwritten Text Recognition (HTR) Engines; with Large Language Models (LLMs) for Historical Document Digitisation*. 10.5281/zenodo.8102666

[CR8] Leifert, G., Romein, C. A., Rabus, A., Ströbel, P. B., & Hodel, T. (2024). *Transkribus and Beyond: Pioneering the Future of Transcription Technology*.

[CR9] Li, M., Lv, T., Chen, J., Cui, L., Lu, Y., Florencio, D., Zhang, C., Li, Z., & Wei, F. (2021). *TrOCR: Transformer-based Optical Character Recognition with Pre-trained Models*. arXiv.Org. https://arxiv.org/abs/2109.10282v5

[CR10] Muehlberger, G., Seaward, L., Terras, M., Ares Oliveira, S., Bosch, V., Bryan, M., Colutto, S., Déjean, H., Diem, M., Fiel, S., Gatos, B., Greinoecker, A., Grüning, T., Hackl, G., Haukkovaara, V., Heyer, G., Hirvonen, L., Hodel, T., Jokinen, M., …, & Zagoris, K. (2019). Transforming scholarship in the archives through handwritten text recognition: Transkribus as a case study. *Journal of Documentation*, *75*(5), 954–976. 10.1108/JD-07-2018-0114

[CR11] Nockels, J., Gooding, P., & Terras, M. (2024). The implications of handwritten text recognition for accessing the past at scale. *Journal of Documentation,**80*(7), 148–167. 10.1108/JD-09-2023-0183

[CR12] Pinche, A., & Stokes, P. (2024). Historical documents and automatic text recognition: Introduction. *Journal of Data Mining & Digital Humanities*, *Historical Documents and...*, 13247. 10.46298/jdmdh.13247

[CR13] Puigcerver, J. (2024). *Jpuigcerver/PyLaia* [Python]. https://github.com/jpuigcerver/PyLaia (Original work published 2017).

[CR14] Rabus, A., & Thompson, W. (2023). Performance of Generic HTR Models on Historical Cyrillic and Glagolitic: Comparison of Engines. *Scripta & e-Scripta,**23*, 11–34.

[CR15] *Republic*. (n.d.). Retrieved 30 September 2024, from https://republic.huygens.knaw.nl/

[CR16] Romein, C. A., Kemman, M., Birkholz, J. M., Baker, J., Gruijter, M. D., Meroño-Peñuela, A., Ries, T., Ros, R., & Scagliola, S. (2020a). State of the field: Digital history. *History,**105*(365), 291–312. 10.1111/1468-229X.12969

[CR17] Romein, C. A., Veldhoen, S., & de Gruijter, M. (2020b). *Entangled histories ordinances low countries.* Zenodo. 10.5281/zenodo.3567844

[CR18] Romein, C. A., Veldhoen, S., & de Gruijter, M. (2020c). *The datafication of early modern ordinances*. *2*. Retrieved September 30, 2024, from http://journal.dhbenelux.org/journal/issues/002/article-23-romein/article-23-romein.pdf

[CR19] Sluijter, R., van Koert, R., Baars, M., Swüste, M., van Gent, M., van Gelder, E., Hollestelle, J., Ruigrok, G., Nijenhuis, I., & Oddens, J. (2023). *REPUBLIC PageXML ground truth handwritten resolutions States General* (Version 1.0) . Zenodo. 10.5281/zenodo.7695131

[CR20] Tarride, S., Schneider, Y., Generali-Lince, M., Boillet, M., Abadie, B., spsampsps Kermorvant, C. (2024). *Improving Automatic Text Recognition with Language Models in the PyLaia Open-Source Library* (No. arXiv:2404.18722). arXiv. http://arxiv.org/abs/2404.18722

[CR21] *UAX #15: Unicode Normalization Forms*. (n.d.-a). Retrieved 30 September 2024, from https://unicode.org/reports/tr15/

[CR22] *UAX #15: Unicode Normalization Forms*. (n.d.-b). Retrieved 30 September 2024, from https://unicode.org/reports/tr15/#Normalization_Forms_Table

[CR23] *UAX #44: Unicode Character Database*. (n.d.). Retrieved 30 September 2024, from https://www.unicode.org/reports/tr44/#General_Category_Values

